# MicroRNA-4516 suppresses proliferative vitreoretinopathy development via negatively regulating OTX1

**DOI:** 10.1371/journal.pone.0270526

**Published:** 2022-06-30

**Authors:** Shu-I Pao, Le-Tien Lin, Yi-Hao Chen, Ching-Long Chen, Jiann-Torng Chen

**Affiliations:** 1 Department of Ophthalmology, Tri-Service General Hospital, National Defense Medical Center, Taipei, Taiwan; 2 Department of Ophthalmology, Tri-Service General Hospital Songshan Branch, National Defense Medical Center, Taipei, Taiwan; 3 Graduate Institute of Medical Sciences, National Defense Medical Center, Taipei, Taiwan; Northwest University, UNITED STATES

## Abstract

Proliferative vitreoretinopathy (PVR) progression is associated with TGF-β2-induced epithelial–mesenchymal transition (EMT) in retinal pigment epithelial (RPE) cells. In cancer cells, miR-4516 downregulates orthodenticle homeobox 1 (OTX1)-mediated cell invasion. Moreover, OTX1 is shown to be involved in invasion and EMT. The purpose of this study was to assess whether microRNA (miR-4516) suppresses EMT in RPE cells. EMT features were assessed using Western blotting, immunocytochemical staining, scratch-wound healing, modified Boyden chamber assay, and collagen gel contraction assay. For *in vivo* testing, a rabbit model was used, which involved induction of PVR by injection of transfected spontaneously arising RPE (ARPE) cells into the vitreous chamber. The putative target of miR-4516 was identified by luciferase reporter assay. Results showed that TGF-β2-induced transdifferentiation and migration of RPE cells was inhibited by miR-4516 delivery. Overexpression of miR-4516 led to upregulation of zonula occludens-1, downregulation of α-smooth muscle actin and vimentin, and cell contractility—all EMT features—in the TGF-β2-treated ARPE-19 cells. MiR-4516 regulated OTX1 expression negatively by binding to its 3’-UTR. TGF-β2-induced phosphorylated ERK was inhibited in miR-4516-overexpressing ARPE-19 cells. MiR-4516 suppressed experimental PVR *in vitro* and *in vivo*. In conclusion, the overexpression of miR-4516 suppresses TGF-β2-induced EMT in a PVR model, and its role in PVR depends on OTX1/ERK. Further research is needed to develop a feasible treatment method to prevent and treat PVR.

## Introduction

The main pathogenesis of proliferative vitreous retinopathy (PVR) involves epithelial-mesenchymal transformation (EMT) of retinal pigment epithelium (RPE) cells due to disorderly growth factors (e.g. TGF-β) in vitreous humor [1–4]. EMT results in a morphological transition of epithelial cells to a mesenchymal phenotype characterized by (i) reassembly of the radially extending F-actin cytoskeleton into parallel arrays, (ii) recognition of the directional arrangement of cells as a scar in vivo or vortex-like arrangement in vitro, (iii) lack of tight junction proteins critical for maintaining the blood-retinal barrier and (iv) proliferation and expression of smooth muscle actin that contributes to the contraction of fibrotic RPE cells, subsequently leading to *in vivo* retinal detachment as observed in PVR pathology [5, 6]. PVR may occur in patients with traumatic eye injury, intraocular inflammation or post-retinal surgery, including retinal hypothermia surgery, laser retinal surgery, pneumatic retinal surgery, scleral flexion or vitrectomy [7–9]. Currently, surgery is the standard PVR treatment [10]. However, PVR is one of the major postoperative complications of rhegmatogenous retinal detection (RRD), which can lead to retinal reattachment surgery failure [4, 11]. Therefore, the pathogenesis and treatment of PVR have attracted much attention in recent years. To date, the pathogenesis of PVR has not been fully understood.

In PVR, RPE cells and myofibroblasts are the main cell types [12]. RPE is associated with the formation and contraction of PVR membranes [13]. Accumulated evidence suggests that inflammatory mediators (e.g., growth factors and cytokines) in vitreous or subretinal fluid play an important role in the occurrence of PVR [14]. Many growth factors (e.g., transforming growth factor [TGF], tumor necrosis factor [TNF] and fibroblast growth factor [FGF]) and cytokines (e.g. interleukin (IL)-1, -6, -8, -10 and interferon-γ) are up-regulated in PVR patients within the vitreous or the subretinal fluid [1, 15, 16]. In the rabbit model of PVR, both TGF-β1 and TGF-β2 were significantly upregulated in both aqueous humor (AH) and vitreous [17]. Yao et al. [18] states that PVR is associated with TGF-β induced EMT in RPE cells. Recent studies also indicate that TGF-β induces EMT through two different signaling, including Smad and mitogen-activated protein kinase (MAPK) [19–21].

MicroRNAs play important roles in homeostasis and pathogenesis by regulating the expression of ERK- and p38-related genes [22]. Over 2,000 microRNAs involve in cell proliferation, differentiation and signal transduction [23–25]. These microRNAs regulate tumor formation, and are implicated in many human diseases. Therefore, the role of microRNAs as therapeutic targets or disease markers has been an active research topic.

The various microRNAs are thought to act on retinal or RPE cells, which plays important roles in neuroprotection and angiogenesis [26, 27]. Recent studies have reported increasing evidence of miRNAs in the EMT of RPE cells [5, 28, 29]. During TGF-β2 induction of EMT in RPE cells, a total of 304 miRNAs changes were reported, of which 119 were upregulated and 185 were downregulated, indicating that miRNAs may be associated with EMT in RPE cells [30]. In these miRNAs, the expression of miRNA-29b was reduced by more than 80% [30]. Recent studies report that miR-4516 promotes apoptosis [31], predicts poor prognosis of human glioblastoma [32], and may potentially become a molecular target for psoriasis regulation [33]. In particular, miR-4516 inhibits keratinocyte motility by targeting fibronectin/integrin α9 signaling [33]. Additional evidence has revealed that long stress-induced non-coding transcripts 5 (LSINCT5) down-regulate miR-4516 expression, reduce proteasome-mediated high mobility group AT-hook 2 (HMGA2) degradation, and promote EMT activation [34]. MiR-4516 negatively regulates orthodenticle homeobox 1 (OTX1) by binding to its 3’-UTR, resulting in reduction of migration and invasion in pancreatic cancer cells [35]. In colorectal and hepatocellular cancer cells, OTX1 is shown to be involved in invasion, EMT, and ERK/MAPK-mediated migration [36, 37]. Jin et al. [38] demonstrates that low miR-4516 is as an independent risk factor for prognosis of colorectal cancer. Based on these findings, we hypothesized that miR-4516 may be involved in EMT-mediated PVR. However, to date, the role of miR-4516 in the development of EMT-related PVR has not been well studied. Therefore, the present study aimed to investigate whether miR-4516 suppresses EMT in RPE toward the development of EMT-related PVR.

## Materials and methods

### Cell cultures and treatment

The human RPE cell line ARPE-19 was obtained from the American Type Culture Collection (Manassas, VA, USA). ARPE-19 cells were cultured in a 1:1 mixture of Dulbecco’s Modified Eagle Medium (DMEM) and nutrient mixture F-12 Ham (Invitrogen-Gibco, Grand Island, NY, USA), which contained 4 mM L-glutamine, to which 10% fetal bovine serum (FBS; Invitrogen-Gibco), 100 U/mL penicillin and 100 μg/mL streptomycin (Sigma-Aldrich, St Louis, MO, USA) were added. The cells were kept in a humidified environment of 5% CO2 at 37°C, and the medium was exchanged twice a week.

ARPE-19 cells were transfected with miR-4516 or miR-negative control (miR-NC; Life Technologies, Carlsbad, CA, USA) for 24 hours, and then with or without 10 ng/mL human recombinant TGF-β2 (PeproTech, New York, NY, USA) for 24 hours extra. Unless otherwise stated, all experiments were performed under serum-free conditions.

### Quantitative Reverse Transcriptase Polymerase Chain Reaction (qRT-PCR)

After removing the medium and rinsing with phosphate buffered saline (PBS) 3 times, total RNA was extracted from the cells using TRIzol reagent (Life Technologies). Next, reverse transcription of an aliquot of 1μgof total RNA using TaqMan MicroRNA Reverse Transcription Kit. Real MOD Probe SF 2X qPCR mix and Step One Plus PCR System (Applied Biosystems, Foster City, CA, USA) were used for quantitative real-time PCR (RT-qPCR). The 2^-ΔΔCT^ method was used to normalize the expression level of miR-4516 to the expression level of control (U6 small nuclear RNA) or miR-361. The expression level of miR-4516 in the presence or absence of 10ng/mL TGF-β2 in RPE cells was also evaluated.

### Western blotting

After the experimental treatments, the cells were lysed and sonicated in lysis buffer (50 mMTris-HCl [pH 7.5], 2% sodium dodecyl sulfate, and 1 mM phenylmethylsulfonyl fluoride). The protein content was quantified using the Pierce BCA protein assay kit (Rockford, IL, USA). Specifically, 20 μg of protein in each sample was loaded and subjected to sodium dodecyl sulfate–polyacrylamide gel electrophoresis, and then transferred to a polyvinylidene fluoride membrane (Millipore, Billerica, MA, USA). After blocking with 5% skimmed milk dissolved in a phosphate buffer solution with Tween20 at room temperature for 1 hour, the membranes were directly probed with primary antibodies against α-smooth muscle actin (α-SMA; Sigma-Aldrich, St. Louis, MO, USA), anti-zonula occludens (ZO)-1 (Zymed Laboratories, South San Francisco, CA, USA), phosphorylated (p)-ERK1/2 (1:1000 dilution), ERK1/2 (1:1000 dilution), and glyceraldehyde 3-phosphate dehydrogenase (GAPDH; 1:25,000dilution) overnight at 4°C and then incubated with the horseradish peroxidase–conjugated secondary antibody (Jackson Immuno Research Laboratories, West Grove, PA, USA) for 1 hour. Next, the proteins were visualized using enhanced chemiluminescence (ECL; Millipore). The Western blotting images were acquired using the UVP BioSpectrum 500 imaging system (Wolflabs Ltd., Pocklington, England) and examined using Vision Works LS Analysis software (Upland, CA, USA). The expression level of GAPDH was used as a loading control.

### Immunocytochemistry

The ARPE-19 cells were seeded into 8-well cell chamber slides and transfected with miR-4516 or miR-NC. Next, they were incubated for up to 24 h in the presence or absence of TGF-β2. After they were washed three times with PBS, the cells were fixed with 4% paraformaldehyde and then treated with 0.1% Triton X-100 for 10 min on ice. They were then incubated with 5% bovine serum albumin in PBS for 1 h at room temperature. Anti-ZO-1 (1:200dilution; Zymed Laboratories) and anti-vimentin antibodies (1:200 dilution; Santa Cruz Biotechnology) were used as the primary antibodies. The secondary antibodies comprised DyLight 488 anti-rabbit immunoglobulin G (IgG) and DyLight 594 anti-mouse IgG antibodies (1:200 dilution; Bethyl Laboratories, Montgomery, TX, USA), respectively. The nuclei were counterstained with 4’,6’-diamidino-2-phenylindole (DAPI) (Sigma-Aldrich). The preparation was fixed with 70% glycerol and examined using a fluorescence microscope (CKX41, Olympus Corporation, Tokyo, Japan).

### Scratch wound healing assay

A modified *in vitro* scratch test was used to assess cell migration *in vitro*. In short, 95% confluent ARPE-19 cells were serum starved for 24 hours and pretreated with 10 μg mitomycin-C for 2 hours to suppress cell proliferation. A sterile 200 μL pipette tip was then used to inflict scratches on the monolayer. Next, the medium was removed and replaced with fresh serum-free medium containing the test substance (TGF-β2, miR-4516, or miR-NC) or control. The Olympus IX70 microscope equipped with a digital camera was used to photograph selected areas at 4x magnifications of 24 and 48 caps. The width of the scratch was measured using Image J software to calculate the distance between its two edges. The wound healing at the central observation area were quantified at 24 and 48 hours by Image J software.

### Cell migration assay

The modified Boden chamber assay was also used to assess cell migration. In short, ARPE-19 cells were seeded in the upper chamber of a 24-well plate coated with fibronectin at a density of 5×10^4^ cells/well (Corning Inc., Corning, NY, USA) with a pore size of 8 μm (ARPE-19 cells could pass this pore size). The lower chamber was inoculated with 0.1% FBS-DMEM-F12 containing 10 ng/mL TGF-β2. After 5 hours of incubation, the cells that migrated to the lower side (insert) were washed with PBS, fixed with cold methanol (4°C) for 10 minutes, and then counterstained with crystal violet (Thermo Fischer Scientific) for 20 minutes. The numbers of migrated cells were counted using a phase contrast microscope. Each insert counts four randomly selected fields.

### Collagen gel contraction assay

Some modifications were made to evaluate collagen gel shrinkage. In short, rat tail type I collagen (Sigma-Aldrich) was dissolved in 0.1% acetic acid in sterile distilled water and stored at 4°C overnight. The 24-well plate was coated with 2% FBS overnight to block non-specific binding. ARPE-19 cells (1.0×10^6^ cells/mL) were resuspended in serum-free DMEM-F12. The cell suspension was mixed with 5.0 mL of 3 mg/mL type I collagen, 3.0 mL of concentrated serum-free DMEM-F12 containing glutamine and antibiotics, and 391 μL of 1 mM NaOH. Next, 350 μL of the mixture was added to each FBS-coated well and solidified by incubating in 5% CO2 at 37°C for 1 hour. After 1.5 hours, the collagen gel was removed from the bottom. A micro spatula was used to add 1 mL of 10% FBS-DMEM-F12 to the top of each gel. After 24 hours, the medium was removed, the gel was washed with serum-free DMEM-F12, and incubated in serum-free DMEM-F12 containing 10ng/mL TGF-β2 at 37°C for another 3 days. The cell culture medium was changed every other day. Collagen gel without RPE cells was used as baseline data for contraction. On the third day, an LAS-3000 Charge Coupled Device Camera (Fujifilm, Dusseldorf, Germany) was used to observe, record and measure the surface area of each matrix. The percentage of gel shrinkage was calculated as follows: [(gel size on day 1-gel size on day 3)/gel size on day 1] × 100. All experiments should be conducted at least 3 times.

### Luciferase reporter assay

The putative binding site of miR-4516 in the 3’-UTR of OTX1 or complementary DNA fragments and the mutant 3’-UTR of OTX1 were amplified and subcloned into the pGL3 luciferase reporter vector (Promega, Madison, Wisconsin, USA). The cells were seeded into a 6-well plate at a density of 1 × 10^5^ cells/well, and then co-transfected with vector (original or mutant 3’-UTR containing OTX1) and miR-4516. After 24 hours of incubation, the cells were collected and prepared with reporter lysis buffer. Next, the dual luciferase reporter gene detection system (Promega Corp, Madison, WI, USA) was used to measure the luciferase activity in each well.

### *In vivo* inhibition of PVR progression by miR-4516 in an animal model

Five New Zealand albino rabbits, each weighing 1.5 kg, were used for the animal model. Basic animal research was approved and performed by the Institutional Animal Care and Use Committee of Taipei National Defense Medical Center, Taiwan (recognized by the International Association for Laboratory Animal Care Evaluation and Certification) (No: IACUC-21-229). The rabbits received humane care as outlined in the “Guidelines for the Care and Use of Laboratory Animals.” The animals were kept under standard conditions (20±11°C, 12 hours light/12 hours dark cycle), with a floor area of 1.5 square feet and a height of 16 inches. A sufficient amount of food and fresh water equipment was provided to accommodate the rabbits’ visits at any time. During the experiment, rabbits were observed daily and checked for activities. All animal experiments were conducted in accordance with the Vision and Ophthalmology Research Association statement on the use of animals in ophthalmology and vision research. After dilation of the pupils by instillation of eye drops containing 5% phenylephrine and 1% tropicamide (2 drops per drop), the rabbits were anesthetized with 50 mg/kg Zoletil and 10 mg/kg xylazine. A 31-guage needle was passed through the sclera 2.5 mm behind the scleral limbus, and 0.1 mL of 10 ng/mL TGF-β2 and 0.1 mL of balanced salt solution (BSS) containing approximately 1 × 10^6^ ARPE-19 cells transfected with miR-4516 or miR-NC was injected directly over the optic disc of the right eye. On days 1, 8, 15, and 22, a total of four doses were administered to each eye.

After the injections were administered, the rabbits underwent indirect ophthalmoscope examination twice a week for 5 weeks (days 1–37). PVR progress was determined using the Fastenberg classification. The staging criteria are listed in S1 Table. After the experiment, the rabbits were euthanized in a CO_2_ chamber. After exposure to CO_2_, each rabbit was placed in room temperature air for 20 minutes to allow for possible recovery.

### H&E staining

All rabbit eyes were re-fixed in neutral buffered formalin, embedded in paraffin, and sectioned (5μm thick). Serial sections closest to the defect center were then collected, floated in a 40°C water bath, placed on organosilane-coated (silylated) microscope slides, and baked overnight at 37°C. The sections were de-paraffinized in xylene and rehydrated in continuous ethanol rinses for hematoxylin and eosin staining.

### Statistical analysis

Data are expressed as mean ± standard error of mean. All experiments were performed in triplicate and repeated at least three times. GraphPad Prism for Windows version 5.01 (GraphPad Software, La Jolla, CA, USA) was used to analyze the data. One-way analysis of variance was performed to determine statistically significant differences, and then Tukey’s post hoc test was performed. A p-value of <0.05 was considered statistically significant.

## Results

### Expression of miR-4516 was suppressed by TGF-β and TNF-α in RPE cells

**Fig 1:**
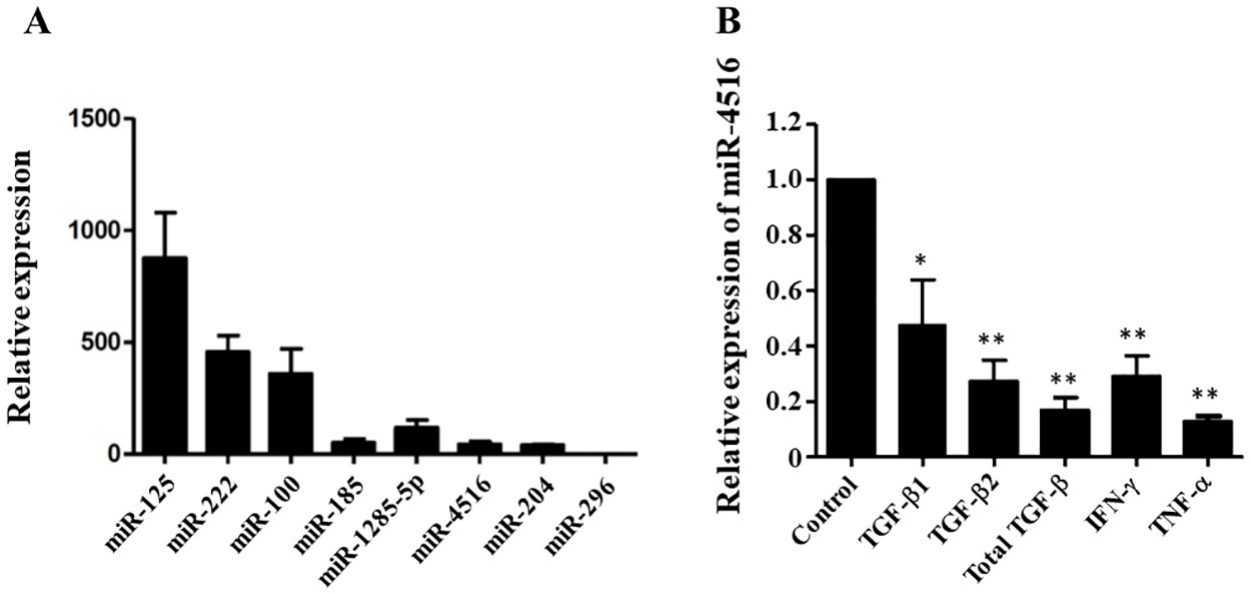
Expression of miR-4516 was suppressed by TGF-β2. (A) Expression of miR-4516 and others in ARPE-19 cells. (B) MiR-4516 expression in ARPE-19 cells treated with or without transforming growth factor (TGF)-β1, TGF-β2, total TGF-β, IFN-γ, and TNF-α (10 ng/mL) for 24 hours. The expression levels of miR-4516 were normalized to those of the control, U6 small nuclear RNA. Data are presented as the means ± standard errors of the mean of three replicates. *p< 0.05; **p<0.01.

To investigate the endogenous expression profile of miR-4516, we first examined the expression of miR-4516 and other well-known microRNAs in RPE cells by qPCR. As shown in Fig 1A, miR-4516 expression is significantly lower than the expression of other well-known microRNAs (miR-125, miR-222 and miR-100). Many inflammatory cytokines, such as TGF-β and TNF-α, act as potent inducers of the EMT in RPE cells, which is implicated in the pathogenesis of PVR [1, 15, 16]. Therefore, we next determined whether these cytokines affected the miR-4516 expression in the RPE cells. By q-RT-PCR, we demonstrated that TGF-β2 (10 ng/mL) and TNF-α (10 ng/mL) significantly inhibited miR-4516 expression in RPE cells (Fig 1B). TGF-β is known to have three isoforms: TGF-β1, TGF-β2, and TGF-β3, among which TGF-β2 is the major isoform. Furthermore, previous study results suggest that TGF-β2 activates other growth factors that contribute to PVR [1, 17]. Therefore, this study used TGF-β2 (10 ng/mL) in the subsequent experiments.

### TGF-β2-induced transdifferentiation of RPE cells was inhibited by miR-4516 delivery

**Fig 2:**
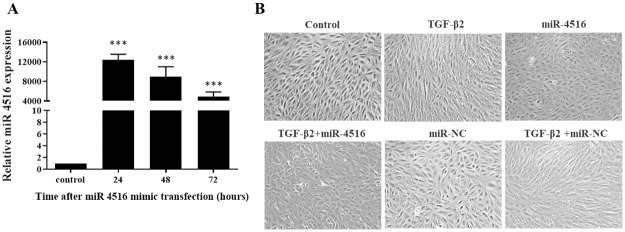
Transdifferentiationin RPE cells was inhibited by miR-4516 delivery. (A) The expression levels of miR-4516 in ARPE-19 cells transfected with negative control or a miR-4516 mimic, as determined through real-time quantitative polymerase chain reaction. ***p<0.001. (B) Morphologic alterations in ARPE-19 cells transfected with negative control or miR-4516 and incubated in the presence or absence of TGF-β2 (10 ng/mL). The cells were examined by phase-contrast microscopy.

RT-qPCR revealed that the level of miR-4516 in the cells was 12,000 times higher than that in ARPE-19 cells transfected with NC at the 24-hour time point. The expression levels decreased gradually over time but remained 4000 times higher than that of cells transfected with NC, even at the 72-hour time point (Fig 2A). We also examined whether miR-4516 is involved in TGF-β2-induced RPE transdifferentiation. As shown in Fig 2B, after treatment of TGF-β2 for 24 hours, fibroblastic transition of the ARPE-19 cells was induced. However, RPE transdifferentiation was significantly suppressed in miR-4516-transfected RPE cells. Cells incubated in the absence of TGF-β2 and transfected with miR-4516 displayed an epithelial morphology.

### Biomarker expression of EMT in TGF-β2-treated ARPE-19 cells through miR-4516 delivery

**Fig 3:**
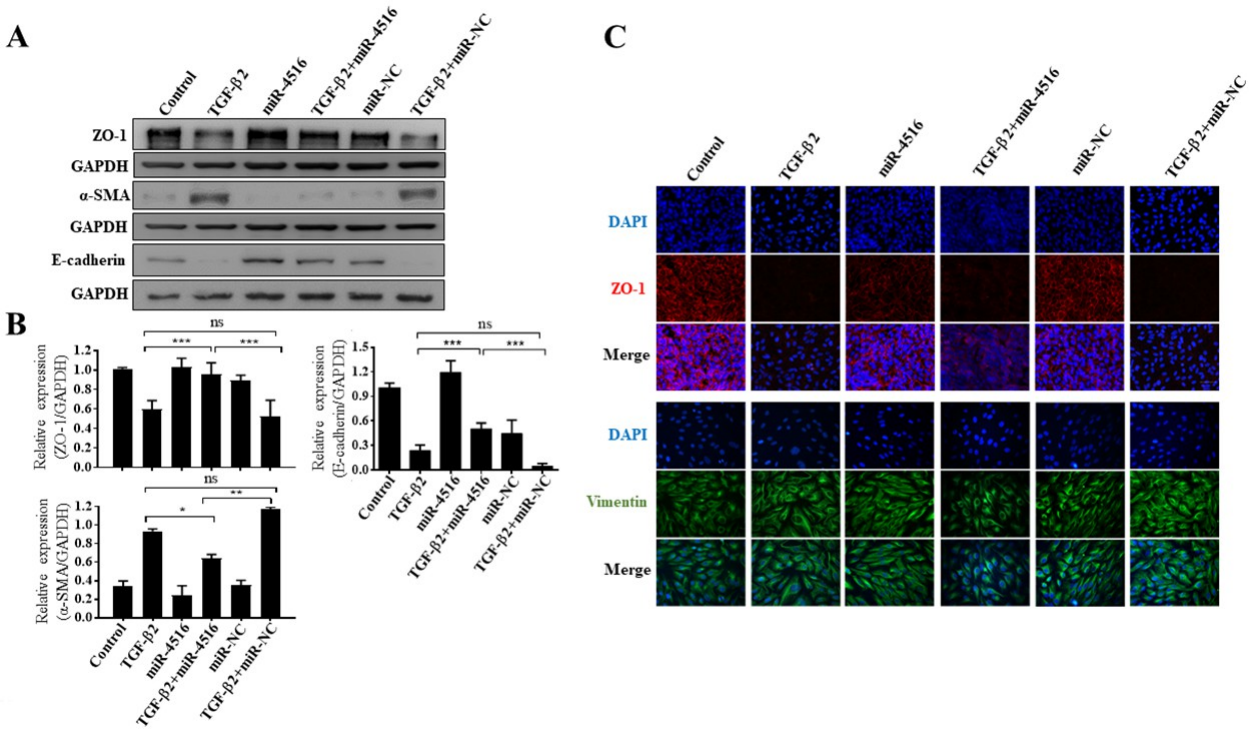
Expression of EMT-related biomarkers in ARPE-19 cells treated with TGF-β2 through miR-4516 delivery. (A) Immunoblot analyses of the effects of miR-4516 overexpression on the expression of α-SMA, ZO-1, and E-cadherin during TGF-β2-induced EMT in the ARPE-19 cells. (B) Quantification of the immunoblot data is shown in (A). Three independent experiments were performed. Data are presented as the means ± standard errors of the mean. *p<0.05; **p<0.01; ***p<0.001; ns: not significant according to an independent one-way analysis of variance. (C) Representative images of immunostained fibroblastic (vimentin) and ZO-1 in ARPE-19 cells transfected with negative control or an miR-4516 mimic and treated with TGF-β2 for 48 h. Nuclei were stained with 4′,6-diamidino-2-phenylindole.

Western blotting was used to examine the protein expression of EMT, including ZO-1, α-SMA, and E-cadherin (epithelial and mesenchymal markers, respectively). As shown in Fig 3A and 3B, quantitative immunoblot analyses revealed that, compared to control group, TGF-β2-treated ARPE-19 cells had significantly higher levels of α-SMA and significantly lower levels of ZO-1 and E-cadherin, representing epithelial proteins. However, compared to TGF-β2 treated cells or cells transfected with NC, cells transfected with miR-4516 after TGF-β2 treatment exhibited reduced α-SMA expression and increased expression of ZO-1 and E-cadherin. Immunocytochemistry analysis—specifically immunofluorescence—was used to examine the effects of transfection of ARPE-19 cells with NC or the miR-4516 mimic on the TGF-β2-induced expression of ZO-1 and vimentin (another mesenchymal marker). Consistent with the Western blotting results, ZO-1 and vimentin expression was lower and higher in TGF-β2 treated cells, respectively, than in the control cells. In contrast, in TGF-β2-treated ARPE-19 cells, cells transfected with miR-4516 mimic downregulated and upregulated vimentin and ZO-1, respectively (Fig 3C). However, the expression of these EMT-related proteins was not affected in cells treated with miR-4516 inhibitor (S1 and S2 Figs). Moreover, miR-4516 expression was lower than the expression of other well-known microRNAs (miR-125, miR-222, and miR-100) (Fig 1A). This indicated that miR-4516 inhibitor did not affect the expression of EMT-related proteins due to the low expression of endogenous miR-4516 in APRE-19 cells. Taken together, these results demonstrate that miR-4516 transfection suppressed TGF-β2-induced EMT in the ARPE-19 cells.

### Migration of TGF-B2-treated ARPE19 is passivated by miR-4516 delivery

**Fig 4:**
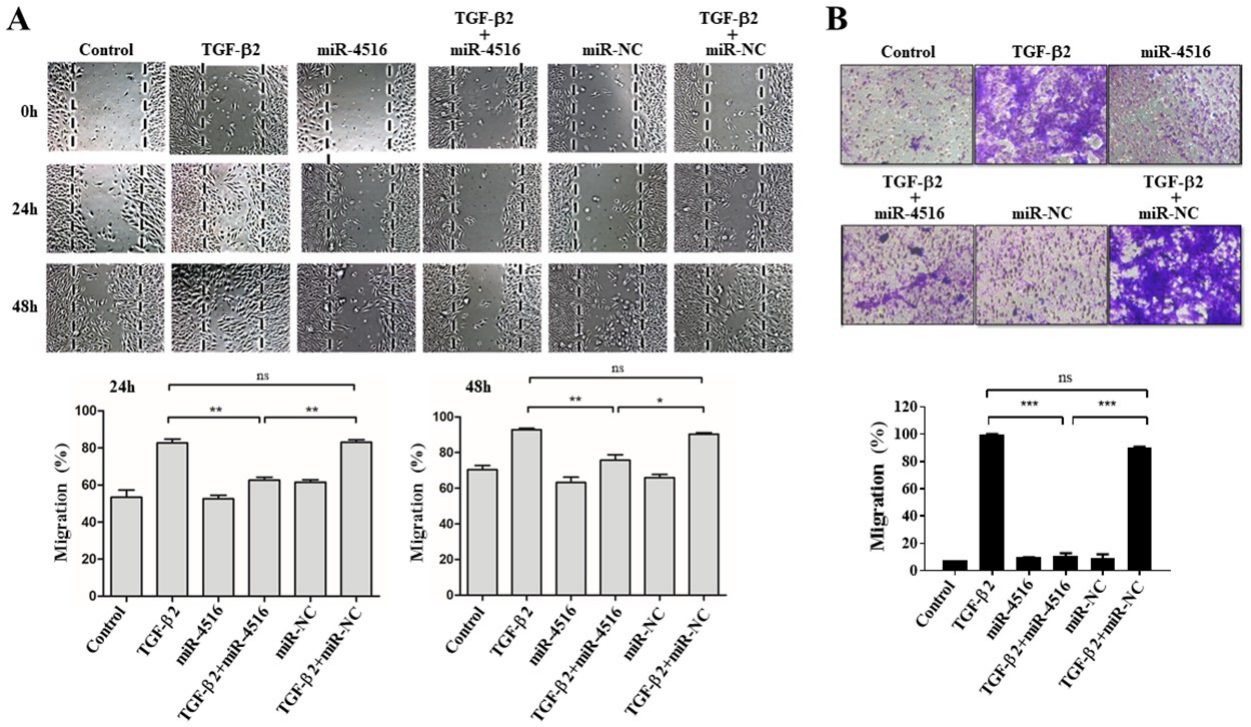
Migration of ARPE-19 cells treated with TGF-β2 was passivated by miR-4516 delivery. (A) Confluent cells were transfected with negative control or a miR-4516 mimic. A scratch was inflicted to the cell surfaces, and then the cells were treated with or without TGF-β2. Light microscopy images were taken at 0, 24, and 48 hours after scratch infliction. The control panel shows the untreated cells. The dashed lines delineate the central observation area and were added by a masked observer to help clarify the extent of migration. Magnification: 4×. The data are representative of at least three independent experiments. The wound healing at the central observation area were quantified at 24 and 48 hours. (B) After the ARPE-19 cells were transfected with the miR-4516 mimic for 24 hours, they were allowed to migrate through the filter of Transwell chambers for 5 hours with or without TGF-β2 (10 ng/mL) and stained with crystal violet. The number of cells that migrated was quantified by counting the number of cells appearing in the lower chamber in four independent vision fields with a 20×microscope objective. Results are presented as the means ± standard errors of the mean of three independent experiments. ***p<0.001; ns: not significant.

TGF-β2-induced EMT in RPE cells is an initiating event in many fibrotic processes, including collagen contraction, cell migration and proliferation, which occur during PVR pathogenesis [14, 18]. Therefore, we studied the effects of miR-4516 transfection on cell migration in TGF-β2-treated ARPE-19 cells using a scratch wound healing assay. Results showed that treatment with TGF-β2 alone enhanced wound closure in a time-dependent manner. At 48 hours, miR-4516 transfection had completely suppressed TGF-β2-induced wound closure (Fig 4A). In sum, miR-4516 transfection inhibited wound closure in TGF-β2-treated ARPE-19 cells. Because cell proliferation and migration are essential to wound closure, we used mitomycin-C to suppress cell proliferation. Therefore, the results suggest that miR-4516 transfection inhibited TGF-β2-induced wound closure by suppressing cell migration (Fig 4A).

Next, we used a modified Boyden chamber method to quantify cell migration in the ARPE-19 cells. As shown in Fig 4B, compared with that in the NC cells, cell migration (purple color) was significantly promoted and suppressed in the ARPE-19 cells, respectively, under TGF-β2 stimulation and miR-4516 transfection (p<0.001). No significant differences were noted between the vehicle control and NC groups. In addition, cell migration was not affected in ARPE-19 cells treated with miR-4516 inhibitor (S3 Fig). In short, the results indicate that miR-4516 transfection inhibited TGF-β2-induced cell migration in the ARPE-19 cells.

### Effects of miR-4516 on EMT are exerted through OTX1

**Fig 5:**
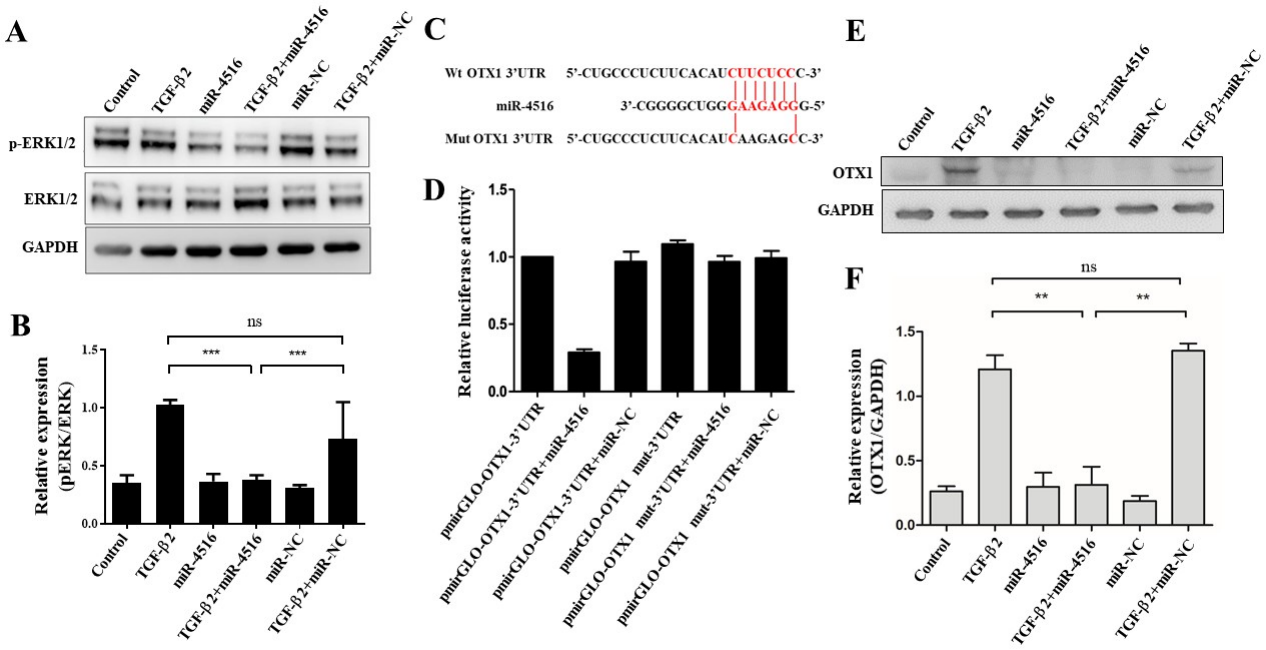
MiR-4516 exerted effects on the EMT through OTX1/ERK signaling pathway. (A) Representative images and quantification of immunostained phosphorylated ERK and total ERK in the TGF-β2-treated ARPE-19 cells transfected with negative control (NC) or miR-4516. Data are presented as the means ± standard errors of the mean of three replicates. Independent one-way analysis was performed, followed by Tukey’s post hoc test. (B) Quantification of the immunoblot data shown in (A). (C) Putative miR-4516 seed matches within the 3’ untranslated region (3’-UTR) of OTX1. The web server TargetScan predicted two perfect seed matches that were conserved across various species. (D) Relative luciferase activity of a pGL3-luciferase reporter vector fused to the native or mutated 3’-UTR of OTX1 in ARPE-19 cells co-transfected with NC or the miR-4516 mimic. (E-F) Western bolt images and quantification of OTX1 in the TGF-β2-treated ARPE-19 cells transfected with negative control (NC) or miR-4516. ***p< 0.001; ns, not significant.

TGF-β transduces EMT through MAPK in RPE cells [20, 21]. Consequently, we evaluated whether miR-4516 regulates TGF-β2-induced EMT via MAPK signaling pathway. As shown in Fig 5A and 5B, after TGF-β2 treatment, p-ERK expression was significantly reduced in miR-4516-transfected RPE cells compared with those transfected with NC. These results were confirmed through quantitative analyses of immunoblotting assays, which indicated that miR-4516 significantly affected p-ERK expression. To identify putative regulatory targets of miR-4516, in silico analysis was performed using the web server Target Scan, which revealed that miR-4516 had the complementary binding sequences targeting 3’-UTR of OTX1 (Fig 5C). Next, luciferase reporter vector containing wild-type (WT) or mutated 3’-UTR of OTX1 was constructed and transfected into RPE cells together with miR-4516 mimics or miR-NC (Fig 5D). As expected, overexpression of miR-4516 specifically inhibited the luciferase activity in cells transfected with reporter vector containing WT 3’-UTR of OTX1, but not with reporter vector containing mutated 3’-UTR of OTX1 (Fig 5D). Subsequent immunoblotting assay also exhibited that miR-4516 significantly inhibited TGF-β2-induced OTX1 expression (Fig 5E and 5F). Taken together, these results indicate that miR-4516 transfection inhibited TGF-β2-induced EMT and PVR development in RPE cells, at least in part, through suppression of the OTX1 signaling pathway.

### PVR is inhibited by miR-4516 overexpression *in vitro* and *in vivo*

**Fig 6:**
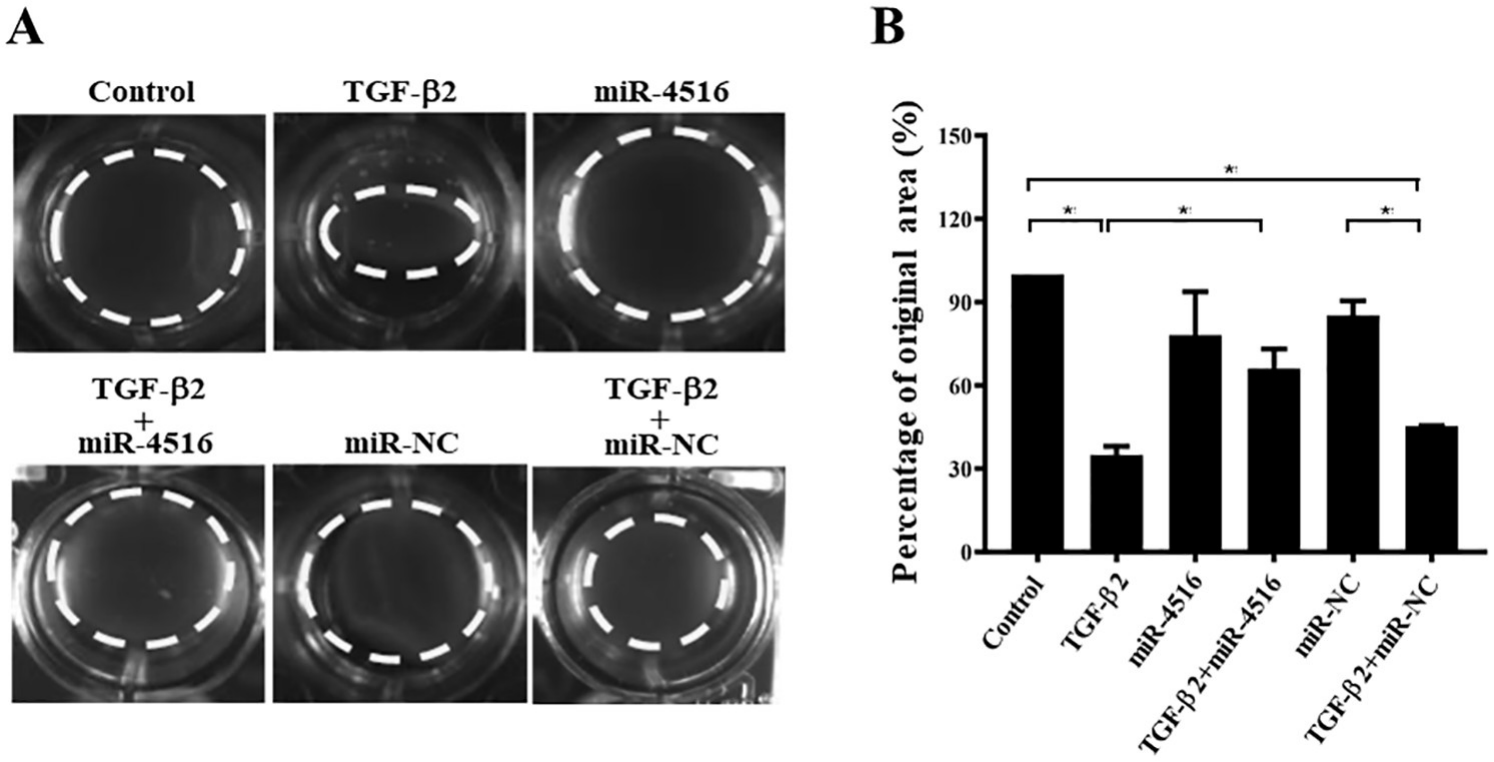
PVR was inhibited by miR-4516 overexpression in vitro. (A) ARPE-19 cells were pre-transfected with negative control or the miR-4516 mimic for 24 hours and then treated with TGF-β2 (10 ng/mL) in the presence or absence of the miR-4516 mimic for 3 days. (B) Contraction or expansion was determined as a percentage of the original area. Results are presented as means ± standard errors of the mean of three independent experiments. *p<0.05; **p<0.01; ns: not significant.

To determine whether miR-4516 regulates TGF-β2-induced PVR of RPE cells, the effects of miR-4516 transfection on collagen gel contraction in TGF-β2-treated ARPE-19 cells were evaluated using a collagen matrix contraction assay. Specifically, freshly polymerized collagen matrices containing ARPE-19 cells were assessed for collagen gel extraction. By collagen gel contraction assay, the area of matrices significantly shrunk in TGF-β2-treated group compared to that in the untreated group (Fig 6). However, this shrinkage was attenuated by the overexpression of miR-4516 (Fig 6), and the development of PVR was not affected in cells treated with miR-4516 inhibitor (S4 Fig). These findings suggest that overexpression of miR-4516 reduced the effect of collagen gel contraction in the TGF-β2-treated RPE cells.

**Fig 7:**
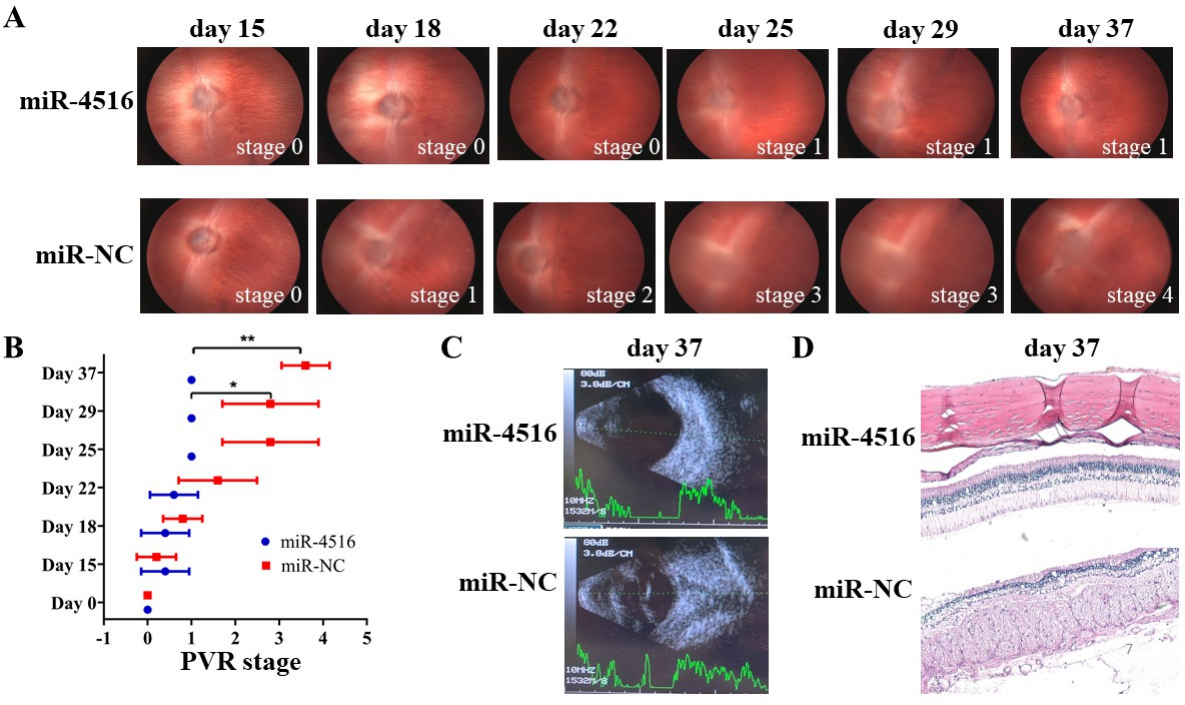
Overexpression of miR-4516 inhibited the PVR progression in vivo. (A) 0.1 mL of TGF-β2 (10 ng/mL) and 0.1 mL of balanced salt solution containing approximately 1 × 10^6^ miR-4516 and ARPE-19 cells transfected with miR-negative control (NC) were injected into the right eyes. A total of four doses was administered to each eye on days 1, 8, 15, and 22. PVR progression was determined according to the Fastenberg classification (stages 1–5) at day 37 post-injection. (B) Results from the independent samples *U* test of differences in PVR stage between the control group and the miR-4516-treated group. (C) Ultrasonic examination of the control eye and the miR-4516-treated eye at day 37 post-injection. (D) Hematoxylin and eosin staining revealed the presence of dense fibrovascular membrane tissue and considerably higher infiltration of inflammatory cells on the retinal surface of eyes treated with miR-NC compared with that of eyes treated with miR-4516. *p<0.05; **p<0.01.

The *in vivo* effect of miR-4516 on PVR development was also examined by injecting 0.1 mL of 10 ng/mL TGF-β2 and 0.1 mL of BSS containing approximately 1 × 10^6^ ARPE-19 cells transfected with miR-4516 and miR-NC into rabbits’ right eyes. A total of four doses were administered to each eye on days 1, 8, 15, and 22. PVR progression was assessed according to the Fastenberg’s classification (S1 Table), which were grouped into 6 stages including stage 0 (without evidence of fibrous ingrowth), 1 (intravitreal membrane), 2 (focal traction; localized vascular changes; hyperemia; engorgement; dilation; blood vessel elevation), 3 (localized detachment of medullary ray), 4 (extensive retinal detachment; total medullary ray detachment; peripapillary retinal detachment), and 5 (Total retinal detachment; retinal folds and holes). As shown in Fig 7A and 7B, in the control group, severe retinal detachment (stages 3 and 4 in the Fastenberg classification) was observed in all rabbits 37 days after injection, although the PVR progress of all rabbits diminished (stage 1 in the Fastenberg classification). S2 Table summarizes the disease status of the rabbits (staged using the Fastenberg classification). As shown in Fig 7B, miR-4516 significantly inhibited PVR development. Ultrasound images revealed severe retinal detachment in the eyes of the control rabbits. The rabbits treated with miR-4516 did not develop this condition (Fig 7C). Hematoxylin and eosin staining showed that compared with the eyes treated with miR-4516, the retinal surface of the eyes treated with miR-NC had dense, fibrous vascular membrane tissue and significantly higher inflammatory cell infiltration (Fig 7D). Compared with eyes injected with ARPE-19 cells transfected with miR-NC, these results show that the progression of PVR in eyes injected with ARPE-19 cells transfected with miR-4516 was significantly inhibited, which indicates that miR-4516 has the potential to prevent and treat PVR.

## Discussion

**Fig 8:**
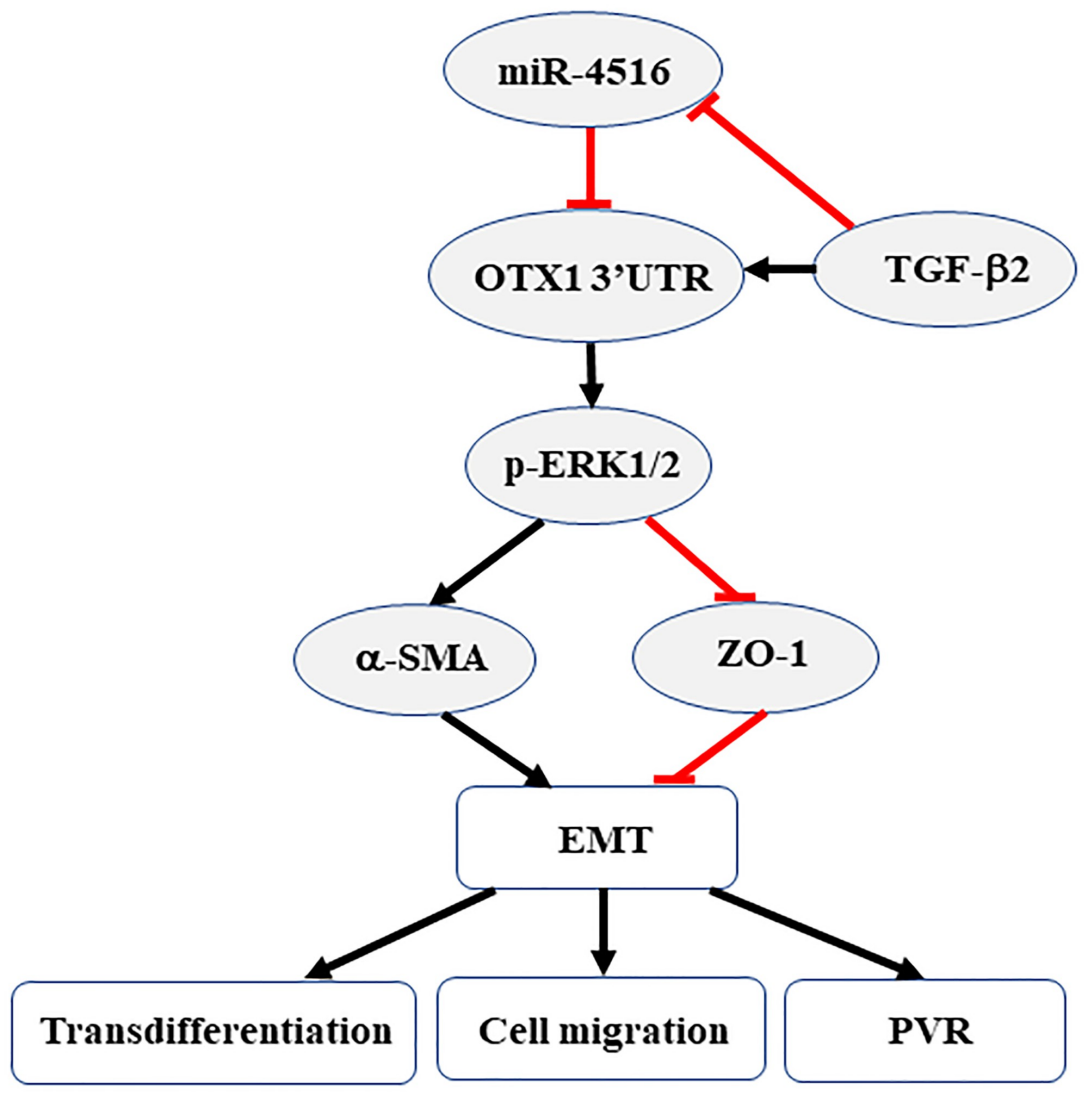
Role of miR-4516 in PVR progression.

In the present study, for the first time, a novel microRNA (miR-4516) was introduced and identified as being involved in the TGF-β2-induced PVR development *in vitro* and *in vivo*. Overexpression of miR-4516 reduced the TGF-β2-induced transdifferentiation and migration in RPE cells. TGF-β2-induced expression of p-ERK and EMT-related proteins were inhibited in RPE cells. In particular, a ERK-related protein, OTX1, was down-regulated in miR-4516-overexpressing RPE cells. Based on these findings, we suggest that overexpression of miR-4516 may down-regulate TGF-β2-induced PVR via the OTX1/ERK/EMT signaling pathway (Fig 8).

In EMT, epithelial cells lose their characteristic epithelial morphology and phenotype and obtain mesenchymal morphology and phenotype [39]. Transdifferentiation is characterized by morphological changes such as decreased cell adhesion and increased cell migration and invasion. Phenotypic changes include decreased expression of epithelial markers such as ZO-1 and E-cadherin, and increased expression of mesenchymal markers such as α-SMA, vimentin, and fibronectin [40]. The essential polypeptide ZO-1 is found in tightly bound complexes, while E-cadherin is found in another type of cell-cell adhesion, adhesion binding. The structure of tight junctions and adherens junctions is important in the structure and function of normal epithelial cells [40–42]. In mesenchymal markers, α-SMA is an intracellular cytoskeletal contractile protein involved in cell movement. Vimentin is another intracellular cytoskeletal protein that plays a key role in stabilizing cell structure during migration [40]. The present study expanded these findings and further showed that miR-4516, a novel microRNA, reduced TGF-β2-induced transdifferentiation, migration and EMT-related proteins (α-SMA and ZO-1) in RPE cells. These findings imply that miR-4516 may suppress transdifferentiation of RPE cells by regulating epithelial protein and intracellular cytoskeletal contractile protein.

PVR is an eye disease characterized by the formation of a dense fibrosis shrinkage membrane consisting of extracellular matrix (ECM) and various cell types in the vitreous cavity and anterior retinal surface [43]. RPE cells are closely connected pigment cell monolayers and are the most important PVR component [43]. Typically, RPE cells remain stationary and maintain their characteristic morphology and function. The external blood-retinal barrier (BRB) usually prevents fluid from the choroid vessels from entering the retina. In a pathological state called retinal rupture, BRB breaks down and RPE cells are exposed to vitreous bodies containing various growth factors and cytokines. Many growth factors and cytokines, including platelet-derived growth factor, FGF, epidermal growth factor, TGF-β, IL-1, IL-6, IL-8, IL-10 and interferon γ, are strong inducers of EMT and are upregulated in the eyes of PVR [1, 15, 16]. RPE cells activated by growth factors and cytokines subsequently undergo EMT, and the resulting dedifferentiated RPE cells migrate, proliferate, and transform into proliferative migratory spindle cells, resulting in PVR [42–44]. These transdifferentiated spindle cells migrate along the subretinal plane and assume a position of the retina by migrating through retinal fracture. In the present study, overexpression of miR-4516 reduced the effect of collagen gel contraction in the TGF-β2-treated RPE cells. In rabbit models, PVR progression was significantly inhibited in the eyes of miR-4516 cells transfected by injection ARPE-19 cells [14]. Meanwhile, RPE cells undergo EMT, resulting in PVR [42, 43]. Another rabbit animal model study also noted that TGF- β induced EMT was associated with PVR progression [18]. Thus, we speculated that miR-4516 suppresses TGF-β2-induced progression of PVR due to inhibition of EMT, which further suggests that miR-4516 has the potential to prevent and treat PVR.

TGF-β is known to transmit its signal through two main pathways, including Smad-dependent pathway and non-Smad pathway [45]. Typical TGF-β/Smad signaling transmits signals by binding to two related type I and type II transmembrane receptors, which further phosphorylate the receptor-regulated Smad protein-Smad2 and/or Smad3 [45]. Phosphorylated Smads work Smad4 with the co-mediator, then translocate to the nucleus and mediate gene transcription [45]. Deckers et al. reported that small interfering RNA targeting Smad4 have been reported to inhibit TGF- β induced EMT in cancer cells [46]. In our previous study, overexpression of miR-1285 inhibited the TGF-β/Smad4 signaling, which both reduced the expression of mesenchymal markers and inhibited the EMT of RPE cells [47]. In contrast, the present study found that overexpression of miR-4516 significantly reduced TGF-β2-induced expression of phosphorylated ERK in RPE cells. Studies have also shown that downregulation of ERK signaling pathways inhibits proliferation, migration and collagen I mRNA expression in RPE cells [48]. Inhibition of the ERK signaling pathway reveals a promising new perspective on treatment and prevention of early PVR [49]. Given these views of ERK, we speculate that miR-4516 inhibited TGF- β induced PVR may be via regulation of the ERK signaling pathway. In addition, other non-Smad signals are involved in the TGF-β of EMT induced in different types of cells, including ERK, p38, and PI3K/AKT methods [21, 50, 51]. In addition, the non-classical signal p38 and PI3K/AKT pathways crosstalk and integrate with the Smad pathway to adjust to each other [51, 52]. To complicate matters, these non-canonical TGF-β signaling and typical Smad signaling can also be mediated through other signaling pathways, such as Notch pathway [45]. In the present study, we did not evaluate the effect of miR-4516 on TGF-β-related Smad- and PI3K/AKT-dependent pathways in RPE cells. Thus, further investigation is needed to determine whether overexpression of miR-4516 inhibits TGF-β2-induced EMT in RPE cells via Smad- and PI3K/AKT-dependent pathways.

Micro RNA (miRNA), approximately 18 to 24 nucleotides in length, is a small molecule of endogenous non-coding RNA that can act as a post-transcriptional regulator. MiRNAs interact with the 3’ untranslated region (3’-UTR) of their target genes to regulate about 30%-50% of protein-coding genes [53]. Huang et al. demonstrated that miR-1285 inhibits the migration and invasion of pancreatic cancer cells and act as tumor suppressor [54]. Overexpression of Mir-1285 significantly inhibits breast cancer cell proliferation [55]. In our previous study, miR-1285 overexpression decreased the Smad4 3’-UTR activity in RPE cells [47]. In the present study, we performed in silico analysis using the web server Target Scan, which revealed that miR-4516 had the complementary binding sequences targeting 3’-UTR of OTX1, SOX4, GRB2 and ETV4, demonstrating that overexpression of miR-4516 significantly decreased 3’-UTR activity of OTX1 in RPE cells. However, overexpression of miR-4516 did not influence TGF-β2-induced expression of SOX4, GRB2, and ETV4 in ARPE cells, although previous studies have reported that SOX4, GRB2, and ETV4 contribute to EMT in cancer cells [56–59]. In cancer cells, miR-4516 negatively regulates OTX1 [35], and the ability of cell migration and invasion is reduced after down-regulation of OTX1 [60]. When OTX1 expression is silenced, the cell proliferation ability and the expression level of related protein p-ERK are reduced, indicating that the role of OTX1 in cancer cells may depend on the activation of p-ERK [60]. As the integration point of various biochemical signals, ERK is involved in a variety of cellular processes, including differentiation, proliferation, transcriptional regulation, and development [61]. Interestingly, we found that a ERK-related protein, OTX1, can be targeted by miR-4516 in RPE cells, which thereby reduces OTX1 promoter activity. Moreover, decreased levels of the associated molecules N-cadherin and vimentin may explain a decrease in cell metastasis capacity [36, 60], as N-cadherin and vimentin are important molecular markers of EMT. In the present study, the EMT-related markers, including α-SMA, ZO-1 and vimentin, were also regulated by miR-4516 in RPE cells. Based on these findings, we suggest that miR-4516 repression of EMT-related PVR may occur in an OTX1/ERK-dependent manner.

The importance of miRNAs in extracellular space has been confirmed by many studies, and exosome-mediated cell exports of miRNAs are the most important features, as well as their uptake and functional consequences in receptor cells [62–65]. In addition to providing delivery strategies for drugs or RNA therapeutics, exosomal components can also act as biomarkers to aid diagnosis and help determine treatment options and outcomes. Luga et al. [66] observed that exosomes derived from cancer‐associated fibroblasts (CAFs) promoted EMT in recipient breast cancer cells via activating autocrine WNT‐planar cell polarity signaling. Recently, miR-543-enriched exosomes induced EMT in RPE cells, suggesting PVR [67]. In the present study, miR-4516 can be used as a biomarker for EMT-related PVR advances. Previous study has shown that miR-4516 is a circulating miRNA [68]. The level of miR-4516 is about 5 times lower in CAF-derived exosomes than normal fibroblast-derived exosomes and are associated with EMT in these cells [69]. Based on these findings, we suggest that further investigations should be conducted to evaluate the level of miR-4516 in RPE-derived exosomes during PVR development and to determine whether or not it is a potential biomarker for PVR.

## Conclusions

Results of the present study show that miR-4516 acts as a biomarker for PVR progression. Overexpression of miR-4516 suppresses collagen gel contraction, transdifferentiation, migration, and EMT-related proteins in RPE cells, regulated by the OTX1/ERK signaling pathway. Therefore, miR-4516/OTX1/ERK may serve as a novel therapeutic target for miRNA-based therapy in PVR.

## Supporting information

S1 Fig Immunoblot analyses of effects of miR-4516 inhibitor on the expression of α-SMA and ZO-1 in TGF-β2-treated ARPE-19 cells. (TIF)

S2 Fig Representative images of immunostained vimentin and ZO-1 in ARPE-19 cells treated with negative control or an miR-4516 inhibitor and treated with TGF-β2 for 48 h. (TIF)

S3 Fig Effect of miR-4516 inhibitor on migration in TGF-β2-treated ARPE cells. (TIF)

S4 Fig Effect of miR-4516 inhibitor on PVR in vitro. (TIF)

S1 Table Fastenberg classification for PCR stage. (TIF)

S2 Table Summary of the stage of PVR formation. (TIF)

S1 Raw images Fig 3A original western blot. (TIF)

S2 Raw images Fig 5A original western blot. (TIF)

S3 Raw images Fig 5E original western blot. (TIF)

S4 Raw images S1 Fig original western blot. (TIF)
