# Supplementary figures and images for: MicroRNA-4516 suppresses proliferative vitreoretinopathy development via negatively regulating OTX1
Source: PLoS One. 2022 Jun 30;17(6):e0270526. doi: 10.1371/journal.pone.0270526 (PMC9246108; doi:10.1371/journal.pone.0270526)

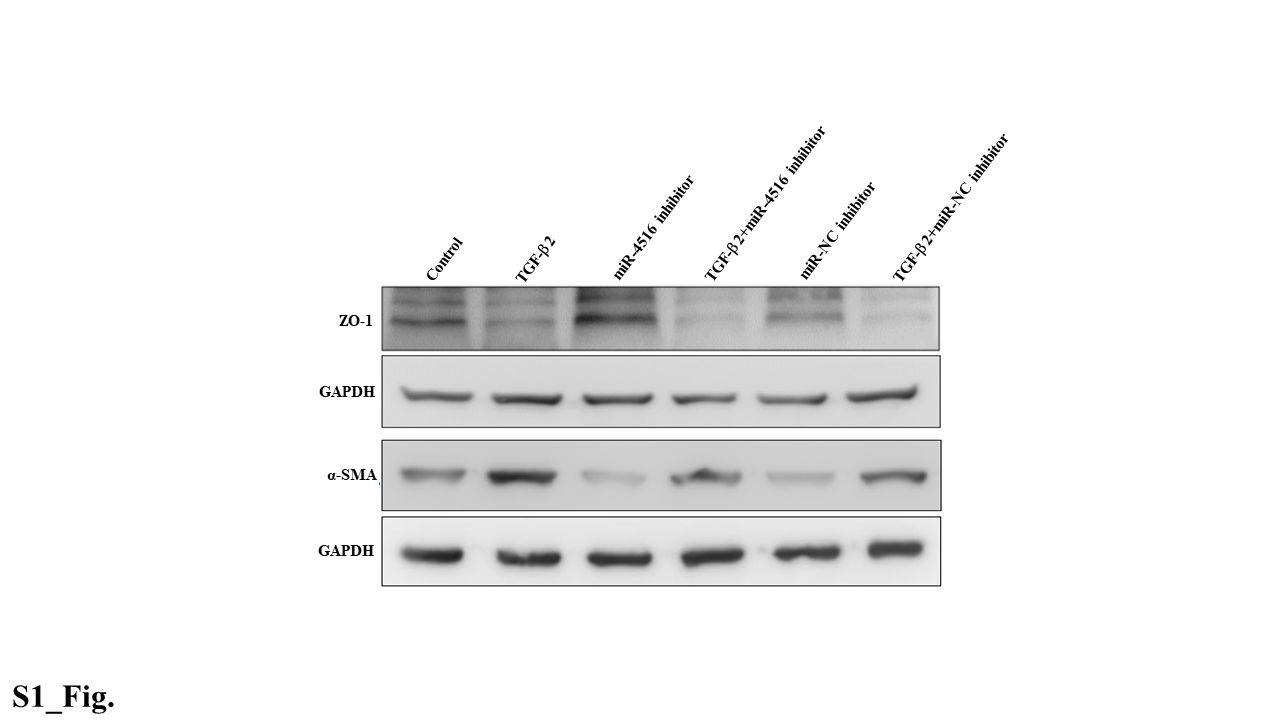

Supplement: S1 Fig — (TIF) [file pone.0270526.s001.tif]

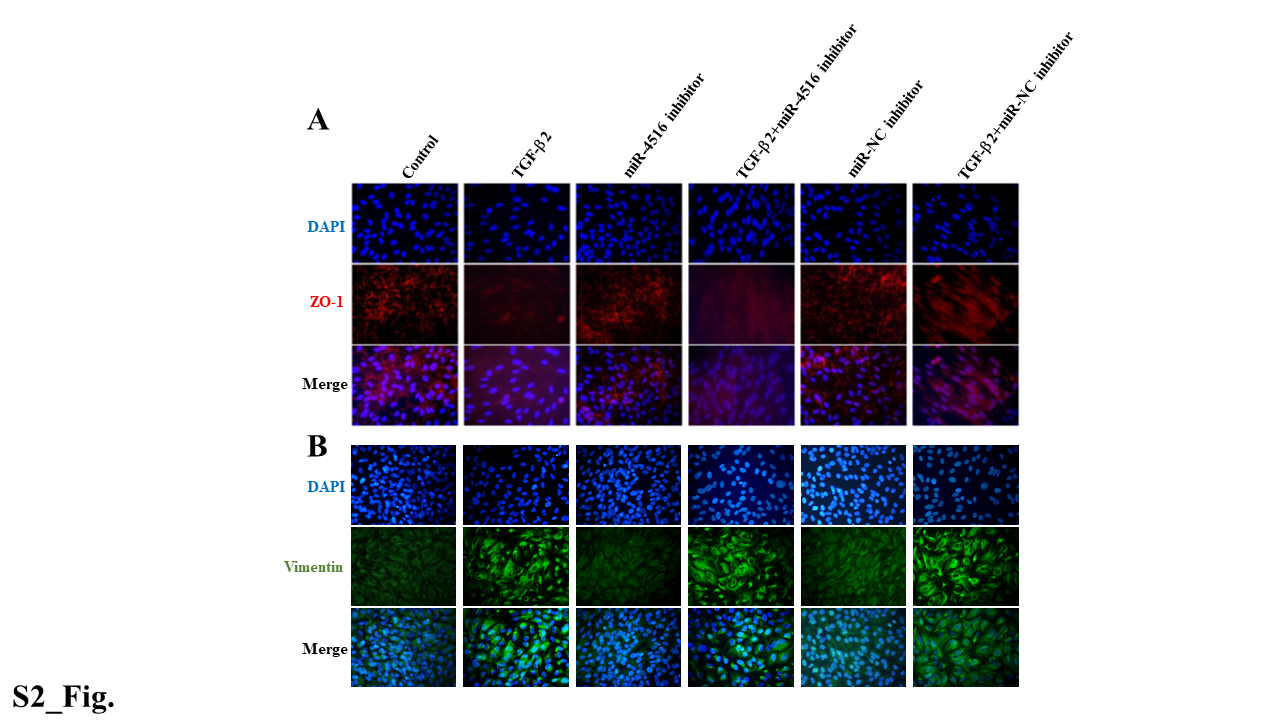

Supplement: S2 Fig — (TIF) [file pone.0270526.s002.tif]

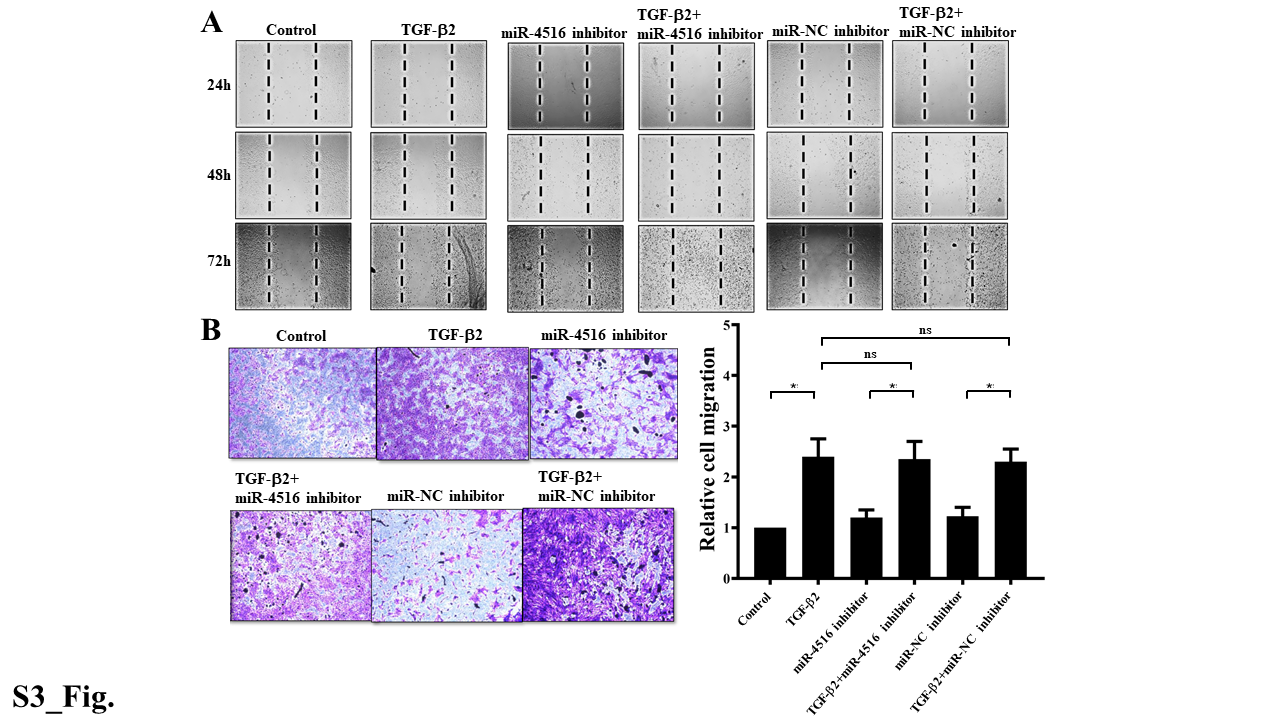

Supplement: S3 Fig — (TIF) [file pone.0270526.s003.tif]

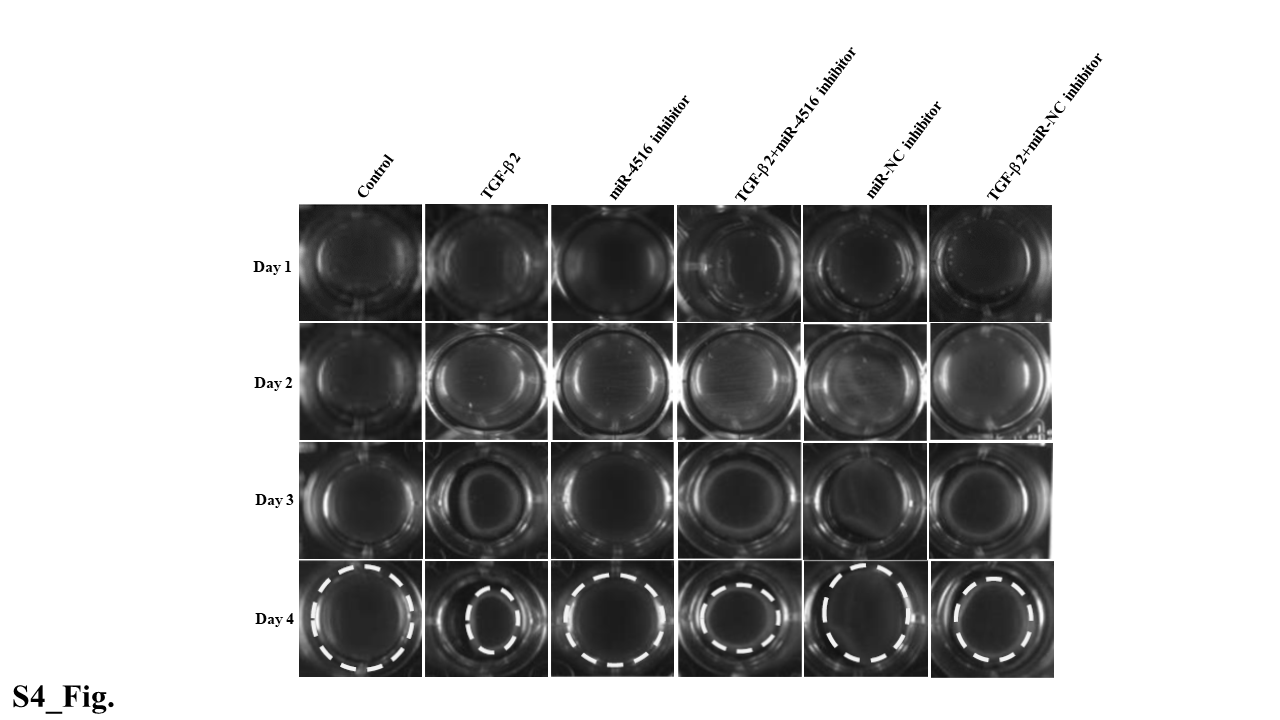

Supplement: S4 Fig — (TIF) [file pone.0270526.s004.tif]

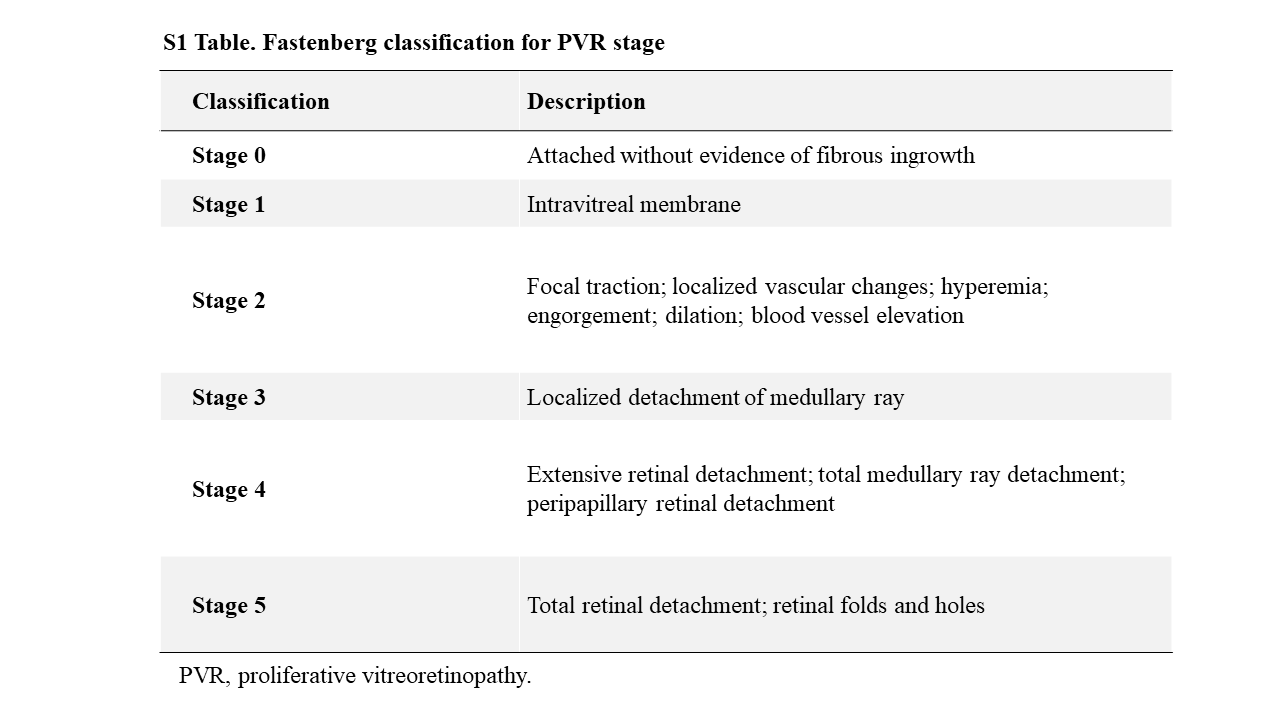

Supplement: S1 Table — (TIF) [file pone.0270526.s005.tif]

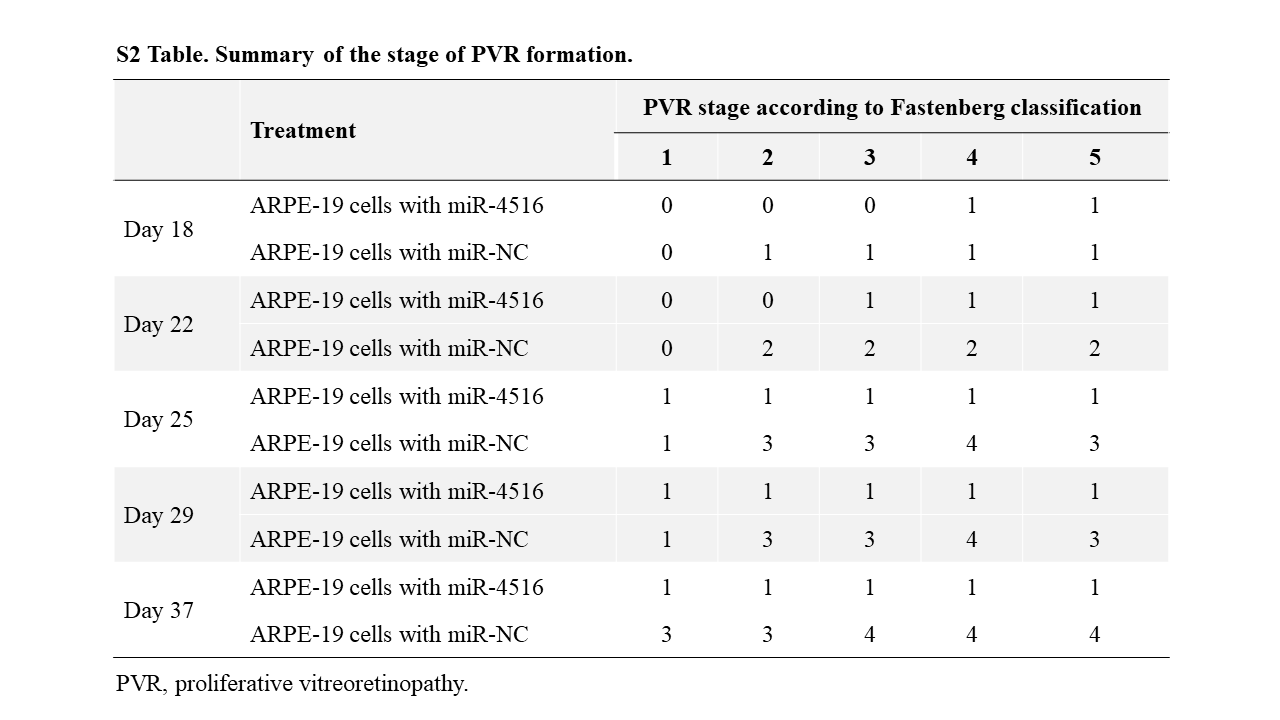

Supplement: S2 Table — (TIF) [file pone.0270526.s006.tif]

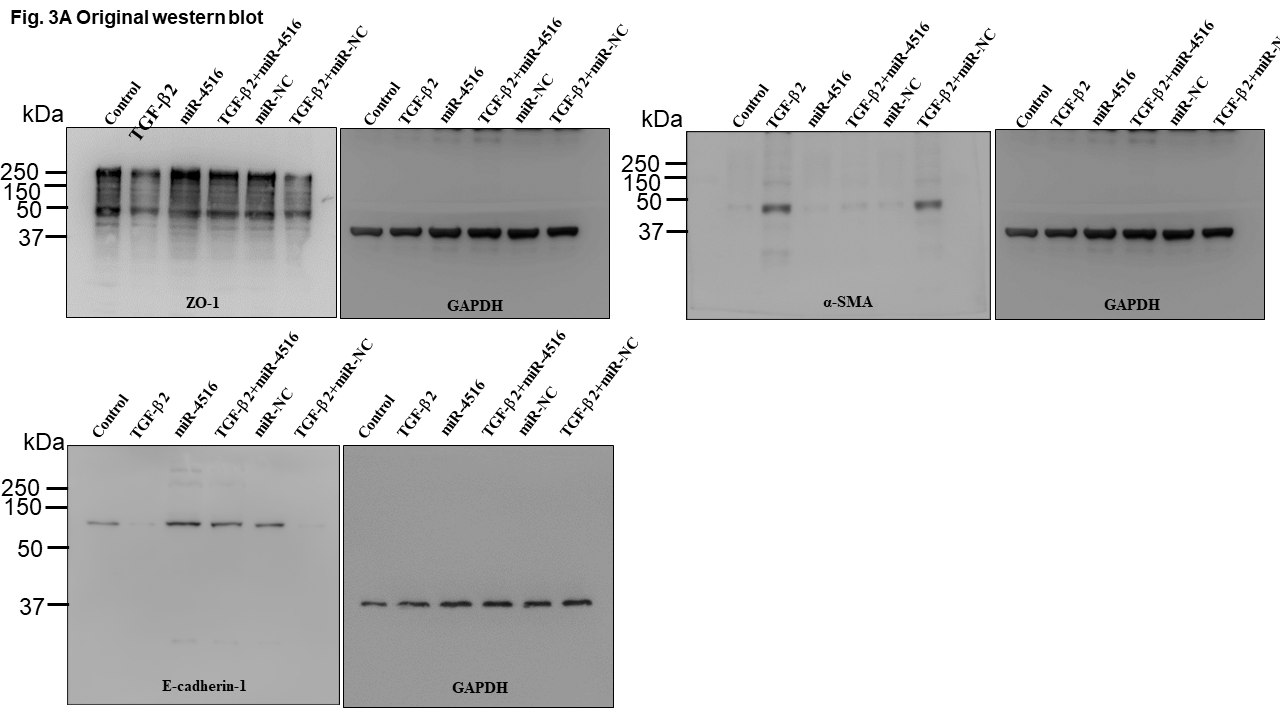

Supplement: S1 Raw images — (TIF) [file pone.0270526.s007.tif]

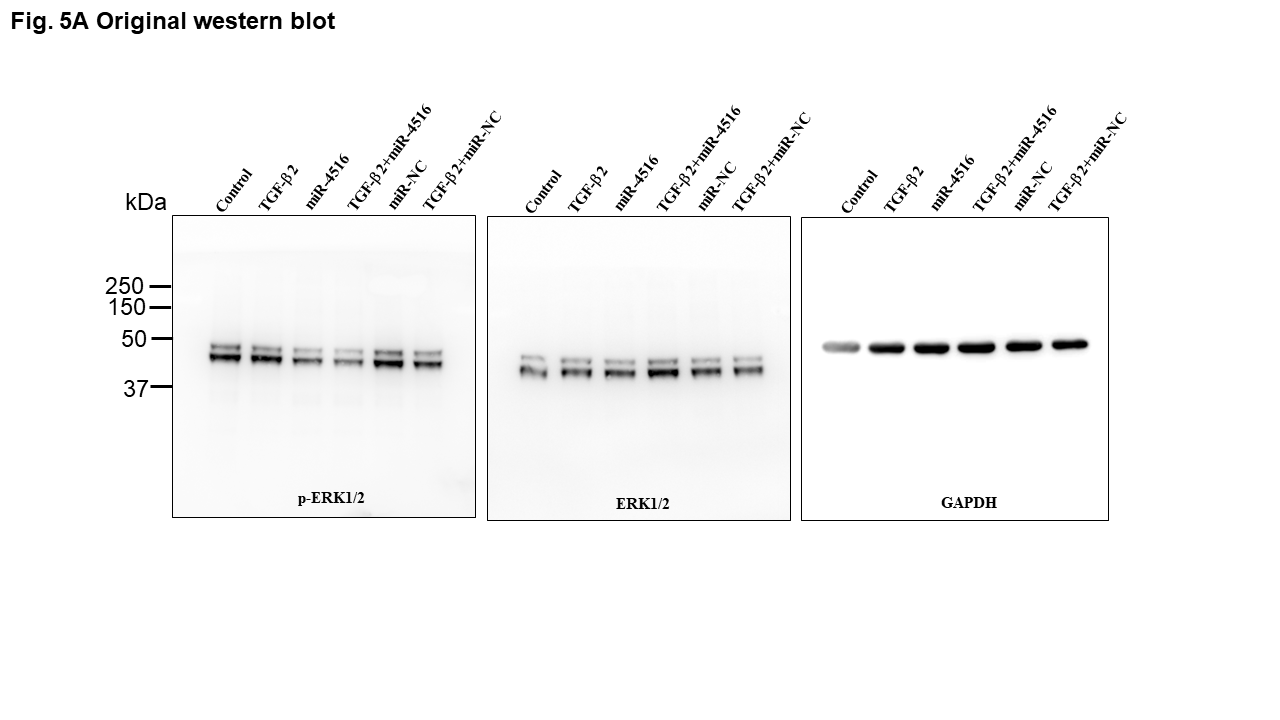

Supplement: S2 Raw images — (TIF) [file pone.0270526.s008.tif]

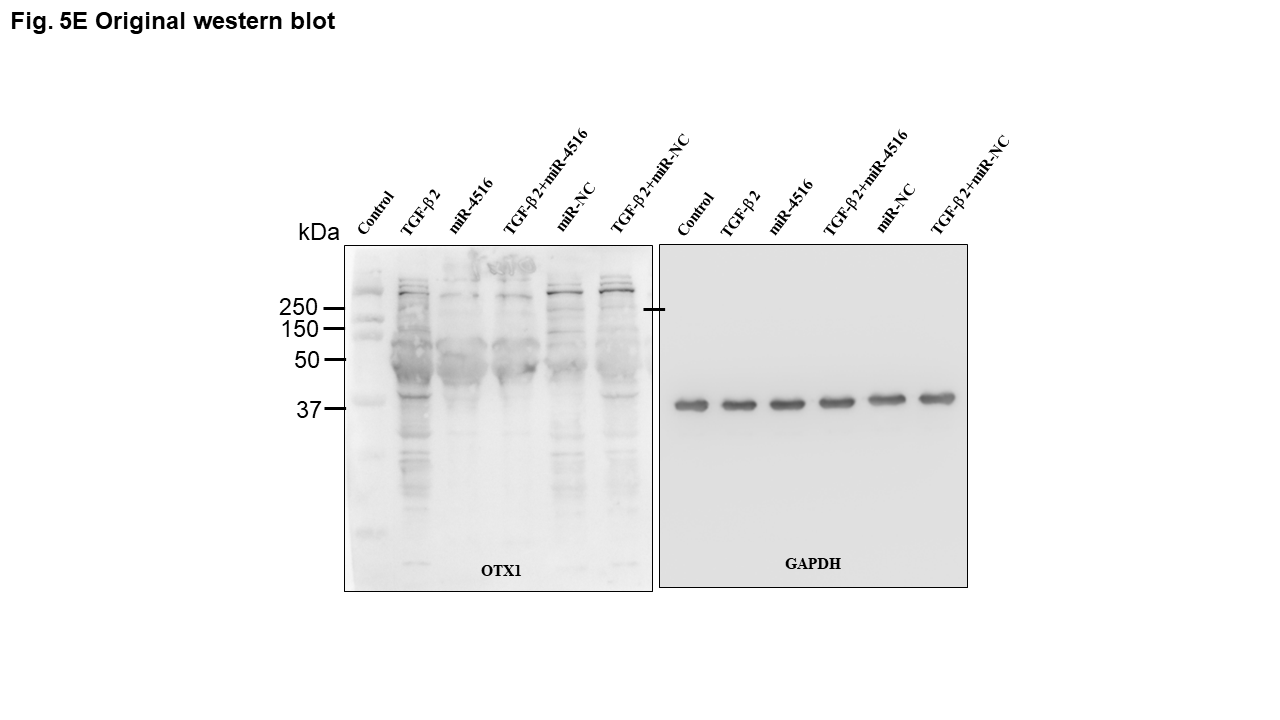

Supplement: S3 Raw images — (TIF) [file pone.0270526.s009.tif]

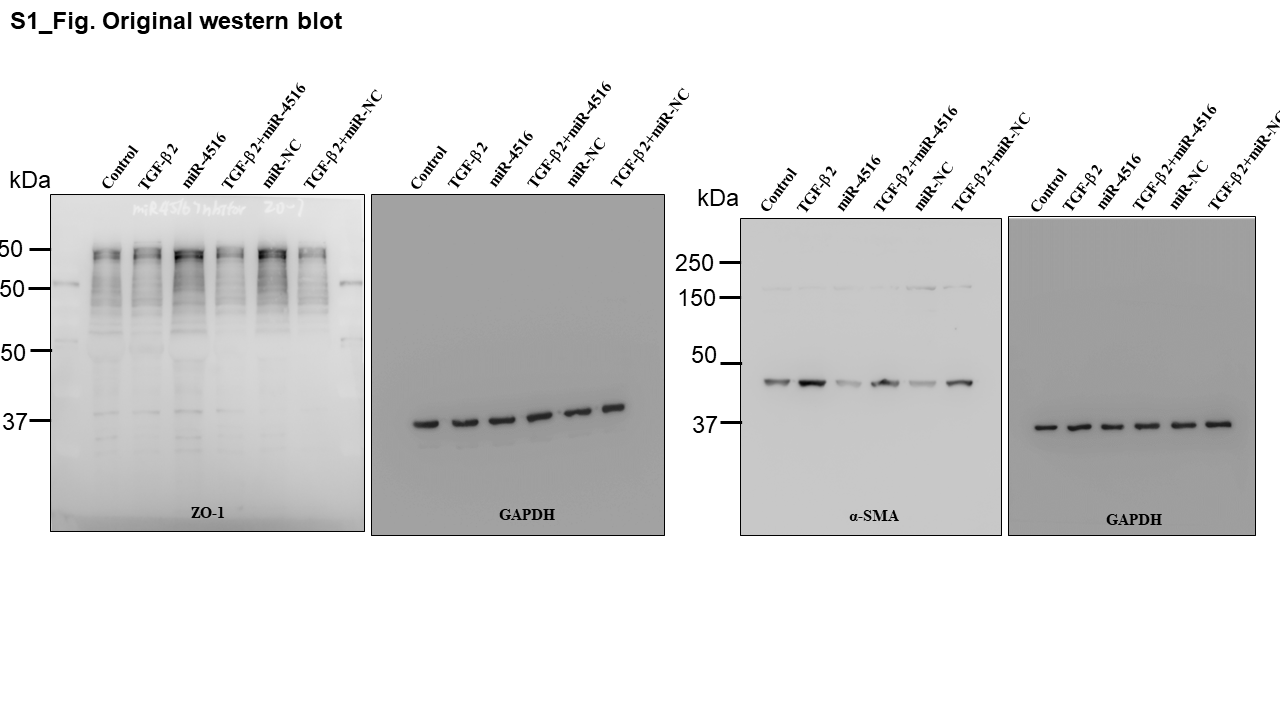

Supplement: S4 Raw images — (TIF) [file pone.0270526.s010.tif]
